# A Dual-Color Luciferase Assay System Reveals Circadian Resetting of Cultured Fibroblasts by Co-Cultured Adrenal Glands

**DOI:** 10.1371/journal.pone.0037093

**Published:** 2012-05-15

**Authors:** Takako Noguchi, Masaaki Ikeda, Yoshihiro Ohmiya, Yoshihiro Nakajima

**Affiliations:** 1 Health Research Institute, National Institute of Advanced Industrial Science and Technology (AIST), Ikeda, Osaka, Japan; 2 Molecular Clock Project, Research Center for Genomic Medicine, Saitama Medical University, Hidaka, Saitama, Japan; 3 Department of Physiology, Saitama Medical University, Moroyama, Saitama, Japan; 4 Bioproduction Research Institute, National Institute of Advanced Industrial Science and Technology (AIST), Tsukuba, Ibaraki, Japan; Karlsruhe Institute of Technology, Germany

## Abstract

In mammals, circadian rhythms of various organs and tissues are synchronized by pacemaker neurons in the suprachiasmatic nucleus (SCN) of the hypothalamus. Glucocorticoids released from the adrenal glands can synchronize circadian rhythms in other tissues. Many hormones show circadian rhythms in their plasma concentrations; however, whether organs outside the SCN can serve as master synchronizers to entrain circadian rhythms in target tissues is not well understood. To further delineate the function of the adrenal glands and the interactions of circadian rhythms in putative master synchronizing organs and their target tissues, here we report a simple co-culture system using a dual-color luciferase assay to monitor circadian rhythms separately in various explanted tissues and fibroblasts. In this system, circadian rhythms of organs and target cells were simultaneously tracked by the green-emitting beetle luciferase from *Pyrearinus termitilluminans* (ELuc) and the red-emitting beetle luciferase from *Phrixothrix hirtus* (SLR), respectively. We obtained tissues from the adrenal glands, thyroid glands, and lungs of transgenic mice that expressed ELuc under control of the promoter from a canonical clock gene, *mBmal1*. The tissues were co-cultured with Rat-1 fibroblasts as representative target cells expressing SLR under control of the *mBmal1* promoter. Amplitudes of the circadian rhythms of Rat-1 fibroblasts were potentiated when the fibroblasts were co-cultured with adrenal gland tissue, but not when co-cultured with thyroid gland or lung tissue. The phases of Rat-1 fibroblasts were reset by application of adrenal gland tissue, whereas the phases of adrenal gland tissue were not influenced by Rat-1 fibroblasts. Furthermore, the effect of the adrenal gland tissue on the fibroblasts was blocked by application of a glucocorticoid receptor (GR) antagonist. These results demonstrate that glucocorticoids are strong circadian synchronizers for fibroblasts and that this co-culture system is a useful tool to analyze humoral communication between different tissues or cell populations.

## Introduction

Circadian oscillations in mammalian clock gene expression are found in various organs and tissues in the body. Various organs show unique phases and periods of circadian rhythms when they are removed from the body and cultured, but the independent oscillators are synchronized by the suprachiasmatic nucleus (SCN) in the hypothalamus, the central clock pacemaker [1]. The SCN output mechanism that controls the circadian rhythms of the whole body includes both neuronal and humoral pathways [2], [3]. SCN neurons project to many other brain regions, such as the subparaventricular zone, the dorsomedial nucleus, and the paraventricular nucleus of the hypothalamus [2]. Other brain regions and endocrine glands directly or indirectly controlled by the SCN release neuropeptides and hormones with diurnal patterns. Hormones with diurnal patterns include corticotropin-releasing hormone, adrenocorticotrophic hormone, thyrotropin, melatonin, glucocorticoids, and gonadotrophins [3]–[6].

As shown in a parabiosis study of intact and SCN-lesioned mice, diffusible signals can regulate circadian rhythms of clock gene expression in some peripheral organs [7]. Among the many hormones that exhibit circadian rhythms in their plasma concentrations, glucocorticoids are considered to be one of the most important synchronizers of various organ clocks in the body [3], [6], [8]. The glucocorticoid hormone analog dexamethasone synchronizes circadian gene expression in cultured Rat-1 fibroblasts and transiently changes the phase of circadian gene expression in the liver, kidney, and heart [9]. A majority of circadian cycling genes identified in the liver lose rhythmicity in adrenalectomized mice [8]. Interestingly, not only SCN rhythmicity but also circadian rhythmicity intrinsic to the adrenal glands is required for circadian glucocorticoid production [10], [11]. There is evidence that other hormones also play an important role in circadian rhythms. For example, gonadectomy affects locomotor rhythms in mice [5]. Diffusible signals directly released from the SCN also regulate circadian rhythms of locomotor activity [12]. To understand how signals initiated by the SCN or other organs synchronize the circadian rhythms of the body, it is important to study the effects of humoral factors on target cells.

The molecular mechanism of the clock is based on multiple transcriptional/translational feedback loops in which BMAL1 and CLOCK drive expression of *Per* and *Cry* genes, causing PERs and CRYs in turn to repress transcription of their own genes [1], [4]. *Bmal1* expression oscillates in cells and tissues and its importance was proven by the fact that deletion of *Bmal1* caused loss of circadian rhythmicity at the whole animal level [13] and the single cell level [14].

Co-culture is an easy and powerful method to study interactions between tissues and cells. Interesting aspects of circadian rhythm interactions have been demonstrated using co-culture. For example, astrocytes co-cultured with an SCN slice exhibited long-lasting gene expression rhythms [15]. Immortalized SCN2.2 cells derived from rat SCN imposed metabolic rhythms on NIH3T3 fibroblasts [16] and PER2 expression rhythms on primary fibroblasts [17].

Luciferases, enzymes that catalyze the emission of light by oxidation of their substrates, luciferins [18], have been utilized in co-culture studies to monitor clock gene expression [15], [17]. Real-time monitoring of luciferase activity in co-cultured living cells or tissues eliminates the need for laborious regular sampling of cells to measure circadian rhythms of clock gene expression. In conventional luciferase systems, however, only one tissue or cell population can be monitored because a single luciferase reporter is used to track clock gene expression. To precisely analyze the interaction between two types of cells or tissues, it is important to monitor them simultaneously.

Recent improvements of luciferases and detection systems have allowed us to monitor the expression of multiple genes simultaneously [19]–[21]. To detect two genes simultaneously, green- and red-emitting beetle luciferases that act on a single _D_-luciferin substrate are commonly used. Any two or more luciferases that stably emit separable emission spectra can be combined to monitor the expression of multiple genes simultaneously [19]–[21]. Mixed emission spectra are measured simultaneously and discriminated with an optical filter. Dual-color luciferase systems have been used for cell extraction assays in mammalian cells [22]–[27]; for cultured plant tissues [28]; and for real-time monitoring in bacteria [29], cultured mammalian cells [30]–[32], and explanted tissues [33], as well as *in vivo* imaging [34]–[36]. These systems can also be applied to monitor gene expression in two co-cultured cell types simultaneously [30]. Application of dual-color luciferase systems in co-culture enables us to observe circadian rhythms of both master organs and target cells and to analyze the effects not only of master organs on target cells, but also of target cells on master organs.

In this study, we co-cultured tissues from different organs (adrenal glands, thyroid glands, and lungs) with Rat-1 fibroblasts as the representative target cells and monitored their circadian rhythms simultaneously. We co-cultured organ tissues from transgenic mice expressing the green-emitting beetle luciferase from *Pyrearinus termitilluminans* (ELuc, λ_max_ = 538 nm) [37], [38] under control of the *mBmal1* promoter (*Bmal1^ELuc^* mice) and Rat-1 fibroblasts expressing the red-emitting beetle luciferase from *Phrixothrix hirtus* (SLR, λ_max_ = 630 nm) [23], [39] under control of the same *mBmal1* promoter (Bmal1-RED fibroblasts) [30].

By examining interactions between the organ tissues and the fibroblasts, we showed that adrenal gland tissue, but not thyroid gland or lung tissue, potentiated the circadian rhythms of fibroblast cultures. Furthermore, a glucocorticoid receptor (GR) antagonist blocked the effect of the adrenal gland tissue. These results suggest that specifically glucocorticoids, but not other hormones produced by the adrenal glands, thyroid glands, or lungs, regulate circadian rhythms of fibroblasts.

## Results

### Tissues of *Bmal1^ELuc^* mice and Rat-1 fibroblasts show distinct circadian rhythms

**Figure 1:**
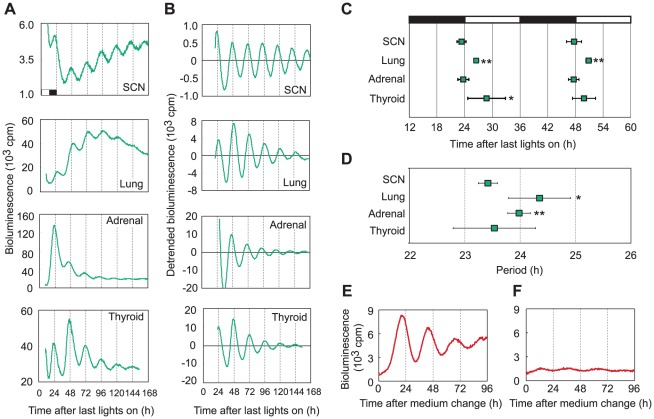
*Bmal1*-driven bioluminescence rhythms of transgenic mouse tissues and fibroblasts. (A) Representative bioluminescence recordings of SCN, lung, adrenal gland, and thyroid gland tissues from *Bmal1^ELuc-A1^* mice. The *x* axis indicates the time after the last lights on. (B) Data shown in (A) were detrended to reduce noise and baseline trends, as described in Materials and Methods. Clear circadian rhythms were observed in each tissue. (C) Average peak times (means ± SD) were plotted against the time after the last lights on. Peaks were determined from the detrended data. (D) Average periods (means ± SD). The average phase and period were determined for SCN (n = 6), lung (n = 10), adrenal gland (n = 10), and thyroid gland (n = 6) tissues. One or two explants were obtained from each animal. (E–F) Representative bioluminescence recordings of Bmal1-RED fibroblasts after dexamethasone treatment (E) and without dexamethasone treatment (F). Open bar, the previous light period. Filled bar, the previous dark period. cpm, counts per minute. Bars indicate SD. *: *P*<0.05, **: *P*<0.01, Kruskal-Wallis *H*-test followed by Mann-Whitney *U*-test with Bonferroni correction. Data were compared with SCN. Adrenal, adrenal gland. Thyroid, thyroid gland.

Before co-culturing tissues and fibroblasts, we measured circadian rhythms in tissues of *Bmal1^ELuc-A1^* mice and Bmal1-RED fibroblasts in separate cultures. As demonstrated in Figures 1A and 1B, cultured SCN, lung, adrenal gland, and thyroid gland tissues showed clear circadian rhythms with baseline fluctuations, consistent with a previous report [33]. Each tissue had its own characteristic phase (Figure 1C) and period (Figure 1D), in agreement with previous reports [33], [40]. Bmal1-RED fibroblasts showed clear circadian rhythms after treatment with 100 nM dexamethasone for 2 h (Figure 1E), as reported previously [30]. However, the higher amplitude was not observed after changing the medium (without dexamethasone treatment) from culture medium to luciferin medium for bioluminescence recording (Figure 1F).

### Adrenal glands induce strong circadian oscillation in fibroblasts

**Figure 2:**
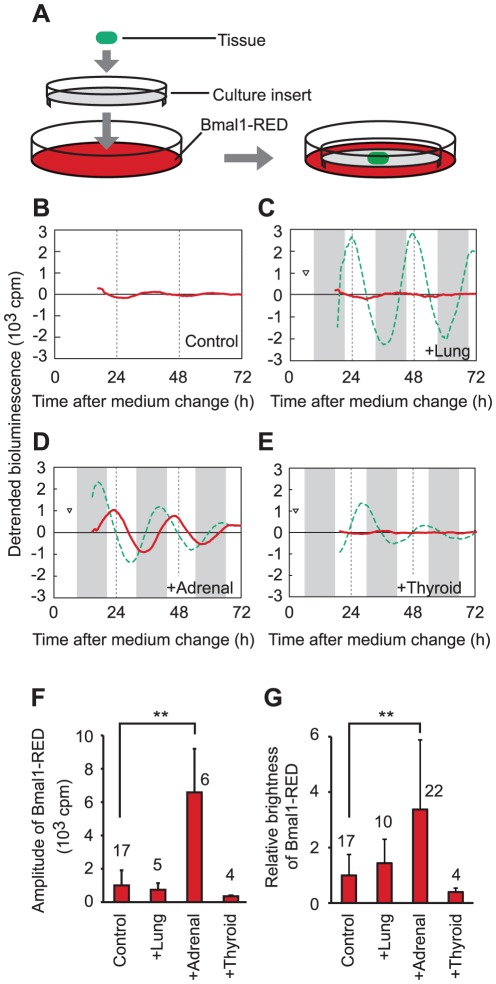
Simultaneous monitoring of *Bmal1* expression in co-cultured tissues and fibroblasts. (A) Schematic diagram of co-culture. Tissues placed on culture inserts were applied to dishes with confluent Bmal1-RED fibroblasts. (B) Representative recording of mono-cultured Bmal1-RED fibroblasts. A circadian rhythm with a small amplitude was induced by the medium change. (C–E) Representative simultaneous bioluminescence recordings of the co-cultured Bmal1-RED fibroblasts (red line) and tissue from *Bmal1^ELuc-A1^* mice (green line). Bmal1-RED fibroblasts were co-cultured with lung (C), adrenal gland (D), or thyroid gland (E) tissue, respectively. Detrended bioluminescence is shown on the *y* axis. The time after the medium change of Bmal1-RED fibroblasts is shown on the *x* axis. Shadings show the projected dark periods for animals. The time when a tissue explant was added to Bmal1-RED fibroblasts is indicated by an arrowhead. (F) Amplitudes of Bmal1-RED fibroblasts in mono-culture (control) or in co-culture with lung, adrenal gland, or thyroid gland tissue. Amplitude was increased in the co-culture with adrenal gland tissue compared to control. Data were obtained from cultures to which tissues were applied within 6 h after the medium change. (G) Relative brightness of Bmal1-RED fibroblasts in mono-culture (control) or in co-culture with lung, adrenal gland, or thyroid gland tissue. Brightness was increased in the co-culture with adrenal gland tissue compared to control. Digits above the columns show the number of samples. Data were obtained from cultures to which tissues were applied 0–24 h after the medium change. Bars indicate SD. **: *P*<0.01, Kruskal-Wallis *H*-test followed by Mann-Whitney *U*-test with Bonferroni correction. Adrenal, adrenal gland. Thyroid, thyroid gland.

In a previous work, we co-cultured two populations of Rat-1 fibroblasts with different periods and found that the circadian rhythms of the two fibroblast populations did not interact significantly [30], consistent with the results of single cell fluorescence [41] or bioluminescence [42] imaging. In this study, we tested whether organs can affect the circadian rhythms of fibroblasts. Tissues from adrenal glands, thyroid glands, and lungs were obtained from *Bmal1^ELuc-A1^* mice and cultured on culture inserts. A culture insert with tissue was placed on a dish with confluent Bmal1-RED fibroblasts within 2 h after changing the medium of Bmal1-RED fibroblasts from culture medium to luciferin medium (Figure 2A). Then, bioluminescence from both tissue and fibroblasts were measured simultaneously. In the mono-cultures, Bmal1-RED fibroblasts did not exhibit a strong circadian rhythm after changing the medium (Figures 1F and 2B). The amplitude was not significantly changed by co-culture with lung tissue (Figure 2C) or thyroid gland tissue (Figure 2E), but was increased 6.6-fold by co-culture with adrenal gland tissue (*P*<0.01; Kruskal-Wallis *H*-test followed by Mann-Whitney *U*-test with Bonferroni correction) (Figures 2D and 2F). The average brightness of Bmal1-RED fibroblasts was also increased 3.4-fold by co-culture with adrenal gland tissue (*P*<0.01; Kruskal-Wallis *H*-test followed by Mann-Whitney *U*-test with Bonferroni correction), but not with the other organ tissues (Figure 2G).

### Adrenal glands reset phase of fibroblasts but fibroblasts do not reset phase of adrenal glands

**Figure 3:**
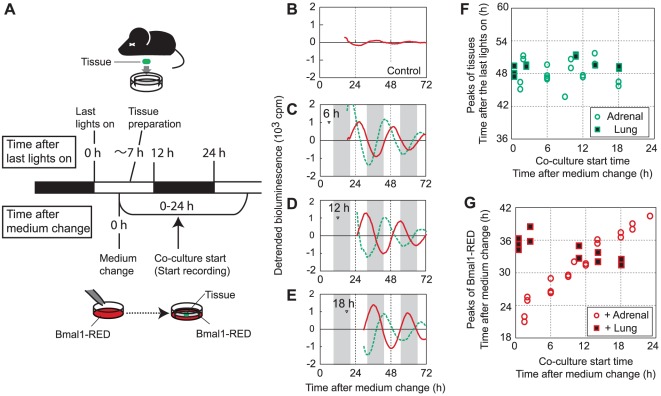
Phase relationship between co-cultured fibroblasts and tissues. (A) Experimental schema. The medium of the Bmal1-RED fibroblasts was replaced with luciferin medium, resetting the phase. Lung tissue, adrenal gland tissue, and thyroid gland tissue were isolated from *Bmal1^ELuc-A1^* mice and cultured in luciferin medium until start of co-culture. At various time points after the medium change on the Bmal1-RED fibroblast culture, a tissue explant on a culture insert was added to the fibroblasts and bioluminescence recordings were started. Open bar indicates light period or projected light period. Black bar indicates dark period or projected dark period. (B) Medium change synchronized the phase of Bmal1-RED fibroblasts and induced a small amplitude rhythm in the cell population. (C–E) Co-culture with adrenal gland tissue induced a large amplitude rhythm in fibroblasts. The peak times of fibroblasts induced by the adrenal gland tissue were independent of the phase of the adrenal gland tissue or the time after the medium change, but were dependent on the co-culture start time. The time of application of adrenal gland tissue is shown by an arrowhead. Bioluminescence recordings of Bmal1-RED fibroblasts and adrenal gland tissue are plotted by red lines and green lines, respectively. Shadings show projected dark periods for animals. (F) Relationship between co-culture start time and phases of tissues. Peak times of adrenal gland tissue (green circles) or lung tissue (green squares) are plotted against co-culture start time. Peak times of both tissues were not affected by the co-culture start time. The co-culture start time after the medium change of Bmal1-RED fibroblasts is shown on the *x* axis. Peak times of tissues after the last lights on for animals are shown on the *y* axis. (G) Relationship between the co-culture start time and phases of Bmal1-RED fibroblasts. Peak times of Bmal1-RED fibroblasts co-cultured with adrenal gland tissue (red circles) or lung tissue (red squares) are plotted against co-culture start time. Co-culture start time on the *x* axis and peaks of Bmal1-RED fibroblasts on the *y* axis were measured relative to the time of the medium change of fibroblasts. Adrenal, adrenal gland. Thyroid, thyroid gland.

Next, we examined how circadian rhythms of tissues and Bmal1-RED fibroblasts affect each other. When Bmal1-RED fibroblasts were cultured without any tissues, weak peaks were apparent at 37.3±2.1 (mean ± SD, n = 16) h after the medium change (Figure 3B). As shown in Figure 1C, when tissues were cultured separately from fibroblasts, 2nd peaks of adrenal gland tissue appeared at 47.6±1.1 (mean ± SD, n = 10) h and 2nd peaks of lung tissue appeared at 50.9±0.6 (mean ± SD, n = 10) h after the last lights on for the animals. To analyze how circadian rhythms of tissues and fibroblasts affect each other, we added tissues (lungs or adrenal glands) to Bmal1-RED fibroblasts at various time points from 0 to 24 h after the medium change of fibroblasts (Figure 3A). In Figures 3C–E, we show representative recordings when adrenal gland tissue was added at 6 h, 12 h, or 18 h after the medium change. If the circadian rhythms of adrenal gland tissue were affected by those of the fibroblasts, the phases of adrenal gland tissue would vary depending on those of the fibroblasts, which was determined by the time of medium change. However, the peaks of adrenal gland tissue appeared around the time of lights on in the previous light-dark cycle (48.1±2.3 (mean ± SD) h after the last lights on) in all cases regardless of fibroblast phase (regression analysis, regression coefficient = −0.03, *P*>0.05). This result indicates that the phase of adrenal gland tissue is not affected by that of fibroblasts (Figure 3F). The peak times of Bmal1-RED fibroblasts were not correlated with the phase of adrenal gland tissue or the time after medium change (Figures 3C–E). The peaks of Bmal1-RED fibroblasts, however, were correlated with the time of start of co-culture with adrenal gland tissue (regression analysis, regression coefficient = 0.82, *P*<0.01) (Figure 3G). The shifted peaks of Bmal1-RED fibroblasts appeared at 20.5±1.7 (mean ± SD) h after the start of co-culture with adrenal gland tissue (Figure 3G). These results indicate that hormonal shock caused by application of adrenal gland tissue shifted the phase of Bmal1-RED fibroblasts. On the other hand, application of lung tissue slightly advanced the phase of Bmal1-RED fibroblasts when the co-culture start time was delayed (Figure 3G). Although the negative correlation between co-culture start time and peaks of Bmal1-RED fibroblasts was significant (regression analysis, regression coefficient = −0.23, *P*<0.01), the change was not as large as that for the application of adrenal gland tissue. Phases of lung tissue appeared at approximately 49.4±1.2 (mean ± SD) h after the last lights on in all cases (regression analysis, regression coefficient = 0.07, *P*>0.05) (Figure 3F). This result indicates that the phase of lung tissue was not significantly affected by the phase of fibroblasts.

### Glucocorticoid receptor mediates transduction of time cue

**Figure 4:**
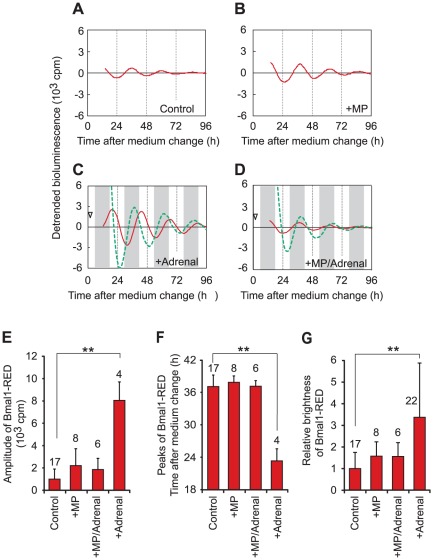
Effects of GR antagonist. Bmal1-RED fibroblasts were cultured without additives (A), with mifepristone (B), with adrenal gland tissue (C), and with both adrenal gland tissue and mifepristone (D). The *x* axis shows the time after the medium change of Bmal1-RED fibroblasts. Shadings show projected dark periods for animals. Bioluminescence recordings from Bmal1-RED fibroblasts and adrenal gland tissue are shown by red lines and green lines, respectively. The times when adrenal gland tissue was applied to Bmal1-RED fibroblast cultures are indicated by an arrowhead. (E) Relative amplitudes of Bmal1-RED fibroblasts with or without mifepristone or adrenal gland tissue. The amplitude of the mono-culture control was used as the standard. Data were obtained from cultures to which adrenal gland tissue was applied within 2.0 h after the medium change. (F) Peak times of Bmal1-RED fibroblasts with or without mifepristone or adrenal gland tissue. Data were obtained from cultures to which adrenal gland tissue were applied within 2.0 h after the medium change. Mifepristone treatment blocked the phase shifts of Bmal1-RED fibroblasts induced by adrenal gland tissue. (G) Relative brightness of Bmal1-RED fibroblasts with or without mifepristone or adrenal gland tissue. Mifepristone treatment blocked the increase in brightness of Bmal1-RED fibroblasts induced by adrenal gland tissue. The brightness of the mono-culture control was used as the standard. Data were obtained from cultures to which tissues were applied 0–24 h after the medium change. Digits above the columns show the number of samples. Bars indicate SD. **: *P*<0.01, Kruskal-Wallis *H*-test followed by Mann-Whitney *U*-test with Bonferroni correction. Adrenal, adrenal gland. MP, mifepristone.

The adrenal glands produce many kinds of hormones. Among the adrenal hormones, glucocorticoids are known to induce synchronized rhythms in fibroblasts [9]. To determine the diffusible factor that potentiates the circadian rhythms of Bmal1-RED fibroblasts, we tested the effect of the GR antagonist, mifepristone. The application of mifepristone produced a slightly higher amplitude in Bmal1-RED fibroblasts, although it was not statistically significant (Figures 4A, B, and E). Mifepristone was applied to Bmal1-RED fibroblast cultures when the medium was changed. Then, adrenal gland tissue was applied to the Bmal1-RED cultures at 0.5–2.0 h after the medium change. Pre-treatment of Bmal1-RED fibroblasts with mifepristone blocked the effect of the adrenal gland tissue on the amplitude of the fibroblasts (Figures 4D and 4E). The peak times of Bmal1-RED fibroblasts with mifepristone treatment alone were compared with those of cultures to which adrenal gland tissue was applied. Phase shifts induced by the adrenal gland tissue were blocked by mifepristone application (Figure 4F). The increase in average brightness of Bmal1-RED fibroblasts induced by co-culture with adrenal gland tissue was also blocked by mifepristone application (Figure 4G). When mifepristone was applied to the co-culture, amplitudes of adrenal gland tissue were decreased by 34% (Figure 4D). However, this effect was not statistically significant (adrenal gland tissue co-cultured with Bmal1-RED fibroblasts without mifepristone, n = 16; with mifepristone, n = 6; Student's *t*-test, *P*>0.05). These results suggest that the time cue signal for Rat-1 fibroblasts from the adrenal glands is mediated by GR.

## Discussion

The present experiments demonstrate that (1) our dual-color luciferase assay system successfully served as a tool to study the relationship between two independent circadian oscillations of co-cultured tissues and cells; (2) adrenal gland tissue is capable of setting the phase and enhancing the amplitude of the circadian rhythms of Rat-1 fibroblasts; and (3) the effects are mediated by GR. Co-culture with thyroid gland or lung tissue did not modulate the circadian rhythms of Rat-1 fibroblasts, although hypothalamic thyroid hormone is suggested to work as a seasonal signal in birds and hamsters [43].

Adrenal explants induced strong oscillations in fibroblasts, which were comparable to the effects of dexamethasone treatment (Figure 2D). Because both amplitude and brightness of fibroblast oscillation increased, both the synchronization of phases of single cells and the induction of higher *Bmal1* promoter activity probably underlay this strong oscillation, as shown in previous reports [44], [45]. The effects of adrenal gland tissue on the amplitudes, phases, and brightness of Bmal1-RED fibroblasts were blocked by application of mifepristone (Figures 4E–G). These results suggest that GR mediates both phase resetting and activation of *Bmal1* expression. The timing of fibroblast rhythms was determined by the time of addition of the adrenal gland tissue to the culture rather than by the phase of the adrenal gland rhythm. This may be because even a small amount of glucocorticoid from adrenal glands exceeds the threshold required to synchronize the circadian rhythms of fibroblasts. Furthermore, glucocorticoid from adrenal glands may have progressively accumulated in the medium under our experimental conditions, suppressing the circadian rhythm of plasma glucocorticoid concentration existing naturally in living animals [3], [10], [46]. Previous reports have demonstrated that adrenal explants were capable of maintaining circadian rhythms of metabolism, glucocorticoid release, and clock gene expression [3], [33], [47], [48]. Use of a perfusion system [49], [50] to more closely mimic the humoral circadian rhythm *in vivo* may allow the demonstration of the entrainment of fibroblasts by glucocorticoids.

The application of lung tissue induced slight but significant phase advances in fibroblasts when the co-culture start time was delayed (Figure 3G). This may indicate that lung-derived cues influenced fibroblast phases; however, we cannot exclude the possibility that mechanical stimulation or temperature changes associated with tissue application caused the phase shifts. Further examination using controls that apply only culture inserts would enable us to distinguish these effects.

In this study, the increase in amplitude of fibroblast rhythms induced by adrenal gland tissue was partially blocked by mifepristone, and the amplitude was reduced to levels similar to those induced by mifepristone alone (Figures 4D and 4E). The slight increase in amplitude induced by mifepristone (Figures 4B and 4E) might be explained by the partial GR agonist activity of mifepristone in some cell types [51]. Although not significant, we also found that the amplitudes of adrenal gland tissue were decreased by mifepristone. It is known that mifepristone can alter adrenal steroidogenesis [52], [53]. It is therefore possible that mifepristone affects clock gene expression. Furthermore, mifepristone works not only as a GR blocker, but also as a progesterone blocker [54]. Because a small amount of progesterone is produced by the adrenal glands, we cannot exclude the possibility that progesterone worked as a time cue and thereby increased amplitudes of Bmal1-RED fibroblasts. However, the importance of glucocorticoid/GR signaling in the circadian system is well known. Mice lacking GR specifically in the liver fail to be entrained by food stimulation [55]. A recent study showed that several canonical clock genes have glucocorticoid receptor response elements in their regulatory regions, and their expression is directly controlled by glucocorticoids [56]. Together with previous studies, the present study supports the idea that glucocorticoids play an important role in synchronizing the circadian rhythms of the body.

In addition to glucocorticoids, the adrenal glands produce hormones, such as mineralocorticoids and androgens from the adrenal cortex and adrenaline (epinephrine) and noradrenaline (norepinephrine) from the adrenal medulla [3], [6]. From the results of this study, we cannot exclude the possibility that other hormones from the adrenal glands contribute to synchronizing circadian rhythms of the whole body, because each hormone has its specific targets, and Rat-1 fibroblasts may not express the receptors for some hormones. For example, the injection of adrenaline/noradrenaline induces *mPer* expression in the mouse liver [57]. Furthermore, there is evidence that other hormones from other organs play an important role in circadian rhythms. For example, gonadectomy affects locomotor rhythm in mice [5], whereas melatonin affects the SCN, the pars tuberalis, and the adrenal glands [58]. Studying the relationships between these humoral factors from major endocrine organs and their specific targets will help us further understand the mechanisms of circadian synchronization of the whole body.

This *in vitro* study demonstrates the important role of glucocorticoids in the resetting of fibroblast circadian rhythms. Further study using dual-color luciferase assay systems in co-culture will help reveal the complex interactions between endocrine glands and their target organs.

## Materials and Methods

### Cell culture and generation of cell line

F2408 (Rat-1) cells (Health Science Research Resources Bank, Osaka, Japan) were grown in culture medium consisting of Dulbecco's modified Eagle's medium (DMEM; Sigma-Aldrich, St. Louis, MO, USA) with 10% fetal bovine serum (FBS; ICN Biochemicals, Aurora, OH, USA), 0.1 mg/ml streptomycin (Nacalai Tesque, Kyoto, Japan), and 100 U/ml penicillin (Nacalai Tesque) at 37°C in a humidified 5% CO_2_ incubator. Bmal1-RED fibroblasts, a Rat-1 cell line that stably expresses SLR (TOYOBO, Osaka, Japan) under control of the *mBmal1* promoter (−816 to +99 bp, where +1 indicates the putative transcription start site) [59], were generated as reported previously [30]. Briefly, the reporter plasmid Bp/915-mRed [22] was transfected into Rat-1 cells using Lipofectamine 2000 (Invitrogen, Carlsbad, CA, USA) with the expression vector for the neomycin resistance gene (pSV-Neo). After transfection for two days, the cells were subcultured for selection with 500 µg/ml geneticin G418 (Nacalai Tesque).

### Generation of transgenic mice

Transgenic mice, *Bmal1^ELuc^* mice, were generated as reported previously [33]. Briefly, the reporter plasmid mBmal1-ELuc carrying the 5′ flanking region (−816 to +99 bp) of *mBmal1* was constructed by replacing the *Nco*I/*Xba*I fragment of Bp/915-Luc [59] with ELuc (TOYOBO) [38]. We generated transgenic mice carrying mBmal1-ELuc from oocytes of BDF1 mice (C57BL/6N Jcl×DBA/2N Jcl, F1) (CLEA Japan, Tokyo, Japan). The linearized DNA fragment (5 ng/µl) was injected into fertilized mouse eggs using standard protocols [60]. Among the 10 transgenic lines of *Bmal1^ELuc^* mice obtained, we selected the *Bmal1^ELuc-A1^* mouse line because it had the most similar bioluminescence intensity to Bma1-RED fibroblasts [30]. *Bmal1^ELuc-A1^* mice were backcrossed to C57BL/6J Jms Slc (Japan SLC, Hamamatsu, Japan) for more than five generations. To simply screen pups for the transgene, a fragment of mouse tail was soaked in 10 µl of luciferin solution (Emerald Luc Luciferase Reagent, TOYOBO) and bioluminescence was measured for 5 s using a luminometer (Phelios AB2350, ATTO, Tokyo, Japan).

### Explant cultures

Mice were maintained in LD12∶12 (lights on: 07.30–19.30 h). During midafternoon (14.00–16.00 h), mice older than 2 months were decapitated. The tissues were rapidly removed and placed in ice-cold Hank's balanced salt solution (HBSS) supplemented with 10 mM HEPES (Sigma), 1.76 mg/l NaHCO_3_ (Invitrogen), 0.1 mg/ml streptomycin, and 100 U/ml penicillin (Nacalai Tesque). The lungs and the adrenal glands were cut into 1–3 mm pieces with a surgical knife. Tissues were cultured on Millicell culture membranes (PICMORG50, Millipore, Billerica, MA, USA). For the thyroid glands, lungs, and adrenal glands, 1.2 ml of luciferin medium consisting of DMEM without phenol red (Gibco, Gaithersburg, MD, USA), 10% FBS, 10 mM HEPES, penicillin/streptomycin, and 200 µM _D_-luciferin potassium salt (TOYOBO) was used. For SCN culture, 1.2 ml of serum-free medium consisting of DMEM (Sigma, D2902), 2% B-27 supplement (Gibco), 10 mM HEPES, 352.5 mg/l NaHCO_3_, 3.5 g/l D-glucose, 200 µM _D_-luciferin potassium salt (TOYOBO), and penicillin/streptomycin was used. Individual tissue cultures were sealed in 35 mm Petri dishes with Parafilm (American Can Co., Greenwich, CT) to prevent evaporation. The procedure adhered strictly to the protocols approved by the Institutional Animal Care and Use Committee of the National Institute of Advanced Industrial Science and Technology.

### Analysis of circadian rhythms in mono-cultured explants

The explants were cultured in the luminometer for more than 6 d to measure their bioluminescence. Raw data collected at 20-min intervals were smoothed using a 3 h moving average [61] and detrended by subtracting a 24 h moving average from the smoothed data. The highest points of the smoothed, detrended data were designated as the circadian peaks. We determined the periods for SCN, lungs, adrenal glands, and thyroid glands from the detrended data in the range 0.5–5.5 d after the start of measurement, applying best-fit cosine curves using the least-squares spectrum method [62].

### Real-time monitoring of bioluminescence

Monitoring of bioluminescence from tissues of *Bmal1^Eluc-A1^* mice and/or from Bmal1-RED fibroblasts was performed with a dish-type luminometer (AB2500 Kronos, ATTO). As in a previous report [30], [33], a luminometer was placed in an incubator set at 20°C and cultures were incubated at 37°C in the luminometer. Bioluminescence was monitored for 1 min at 20-min intervals in the presence or absence of a 620 nm long-pass filter (R62 filter, Hoya, Tokyo, Japan). We expressed the measured bioluminescence intensity as counts per minute (cpm). The activities of ELuc and SLR luciferases were calculated as described previously [22], [30], [33] using the following equation:
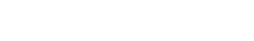
where *ELuc* and *SLR* are *ELuc* and *SLR* luciferase activities, respectively, *F*0 is total counts measured in the absence of the filter, *F*1 is total counts measured in the presence of the filter, and κ*_ELu_*_c_ (0.045) and κ*_SLR_* (0.549) are the filter's transmission coefficients for the respective luciferases.

### Co-culture of explants and fibroblasts

Bmal1-RED fibroblasts (7×10^5^ cells) were seeded in 35 mm Petri dishes and grown to confluence in the culture medium. The culture medium was replaced with 1.1 ml of luciferin medium. An explant from a *Bmal1^ELuc-A1^*mouse cultured on a Millicell culture membrane in another dish was placed in the dish with Bmal1-RED fibroblasts (Figure 2A). Co-culture was started at various time points from 0 to 24 h after the medium change of Bmal1-RED fibroblasts (Figure 3A). Individual dishes were sealed with Parafilm to prevent evaporation and bioluminescence measurement was started. For the experiment shown in Figure 1E, Bmal1-RED fibroblasts were treated with culture medium containing 100 nM dexamethasone (Nacalai Tesque) for 2 h. After dexamethasone treatment, the medium was replaced with luciferin medium for bioluminescence recording. For the GR antagonist treatment, mifepristone (Sigma) was dissolved in DMSO and added to the medium at a final concentration of 10 µM at least 30 min before application of adrenal gland tissue.

### Analysis of brightness, amplitudes, and phases in co-culture

Bioluminescence of the co-cultures was measured for more than 3 d. ELuc and SLR bioluminescence were separated as described above. Brightness of Bmal1-RED fibroblasts was calculated as the average intensity of raw data from 24 to 48 h after the medium change. Co-culture was performed under such conditions that the average intensity of Bmal1-RED fibroblasts was larger than 5% of the brightness of *Bmal1^ELuc-A1^* tissue co-cultured in the same dish, even though our dual-color luciferase assay can detect signals whose intensities are as low as 1% of that of the other mixed color [22]. For amplitude and phase analysis, raw data were smoothed and detrended as described above. The peak in Bmal1-RED fibroblast oscillation appearing 28–52 h after the medium change was defined as peak 1. For adrenal gland tissue, the peak appearing 28–52 h after the last lights on for the animal was defined as peak 1. Amplitudes of rhythms were calculated as the differences in intensity between peak 1 and the previous trough.
